# Piloting a Virtual Graduate Level Course in Translational Geroscience and Precision Gerontology

**DOI:** 10.1093/geroni/igaf122.3826

**Published:** 2025-12-31

**Authors:** Iman Al-Naggar, Ellis Dillon, Christine Thatcher, Richard Fortinsky, David Steffens, George Kuchel

**Affiliations:** UConn Health, Farmington, Connecticut, United States; University of Connecticut, Farmington, Connecticut, United States; UConn Health, Farmington, Connecticut, United States; University of Connecticut, Farmington, Connecticut, United States; University of Connecticut, Farmington, Connecticut, United States; UCONN HEALTH, Farmington, Connecticut, United States

## Abstract

Translational Geroscience is a rapidly growing field of research that seeks to prevent, delay, or reverse multiple chronic diseases of aging simultaneously, thereby increasing the human healthspan, or the years of life free of disease and disability. It aims to do so by targeting the biology of aging, which contributes to loss of resilience and disease onset. Research in Translational Geroscience requires a unique set of skills and has its challenges. Engaging in Translational Geroscience also requires knowledge about issues specific to older adults that are not traditionally covered in clinical and translational research educational programs. As interest in Translational Geroscience grows among clinicians and scientists, the need to train these professionals with various backgrounds has become very clear. To fill this gap, the UConn Older Americans Independence “Pepper” Center (P30AG067988) piloted a virtual, foundational, graduate-level course in Translational Geroscience and Precision Gerontology that was attended by approximately 20 trainees at varying training levels engaged or planning to engage in geroscience research. “Scientific Foundations of Translational Geroscience and Precision Gerontology” was a 34-hour course, divided over 2 semesters (Fall 2023 and Spring 2024) and offered in the late afternoons in 2-hour weekly sessions. Each session was presented by a topic expert who lectured for 40 minutes and took questions for 15 minutes. Two papers per topic, including one comprehensive review, were shared with trainees in advance to read. The course was highly rated by trainees who were engaged and interested, despite difficulties related to the virtual platform.

